# The potential for give and take in plant–microbiome relationships

**DOI:** 10.3389/fpls.2014.00287

**Published:** 2014-06-20

**Authors:** Sarah L. Lebeis

**Affiliations:** Department of Microbiology, University of TennesseeKnoxville, TN, USA

**Keywords:** plant microbiome, massively parallel sequencing, beneficial microbes, microbial communities, plant growth promotion

## Abstract

Mutualistic microbes present in plant-associate microbial communities provide a variety of benefits for their host, including reciprocal exchange of nutrients and/or protection from biotic and abiotic environmental stresses. Plant microbiomes have remarkably robust composition in comparison to the complex and dynamic microbial environments from which they form, suggesting finely tuned discrimination by the plant host. Here the intersection between the plant immune system and microbiomes will be explored, both as a possible means of shaping community membership and as a consequence elicited by certain colonizing microbes. Notably, the advent of massive parallel sequencing technologies allows the investigation of these beneficial microbial functions within whole community settings, so we can now ask how engagement of the immune response influences subsequent microbial interactions. Thus, we are currently poised for future work defining how the plant immune system impacts microbiomes and consequently host health, allowing us to better understand the potential of plant productivity optimization within complex microbial surroundings.

## DETERMINING THE COMPOSITION OF PLANT-ASSOCIATED MICROBIAL COMMUNITIES

Plants have evolved in a microbial world, and, as with many other multicellular organisms, they assemble a specific subset of microorganisms into plant-associated microbial communities both in their aboveground organs (i.e., the phyllosphere) and belowground, inside of the root tissue and in the soil immediately adjacent to and under the influence of the root system (i.e., the rhizosphere). While the mechanisms of immune engagement and suppression during interactions between specific pathogenic and beneficial microbes with their plant host are well-defined, much less is understood about the interactions between the plant and its microbiome as a whole entity. Although microbes living inside of healthy plants are likely to positively influence the host (Weyens et al., 2009), it has been challenging to absolutely define the composition of plant microbiomes. However, new sequencing technologies have opened the door to answer these questions as well as set the stage to understand the mechanisms of microbiome assembly and functions.

**FIGURE 1:**
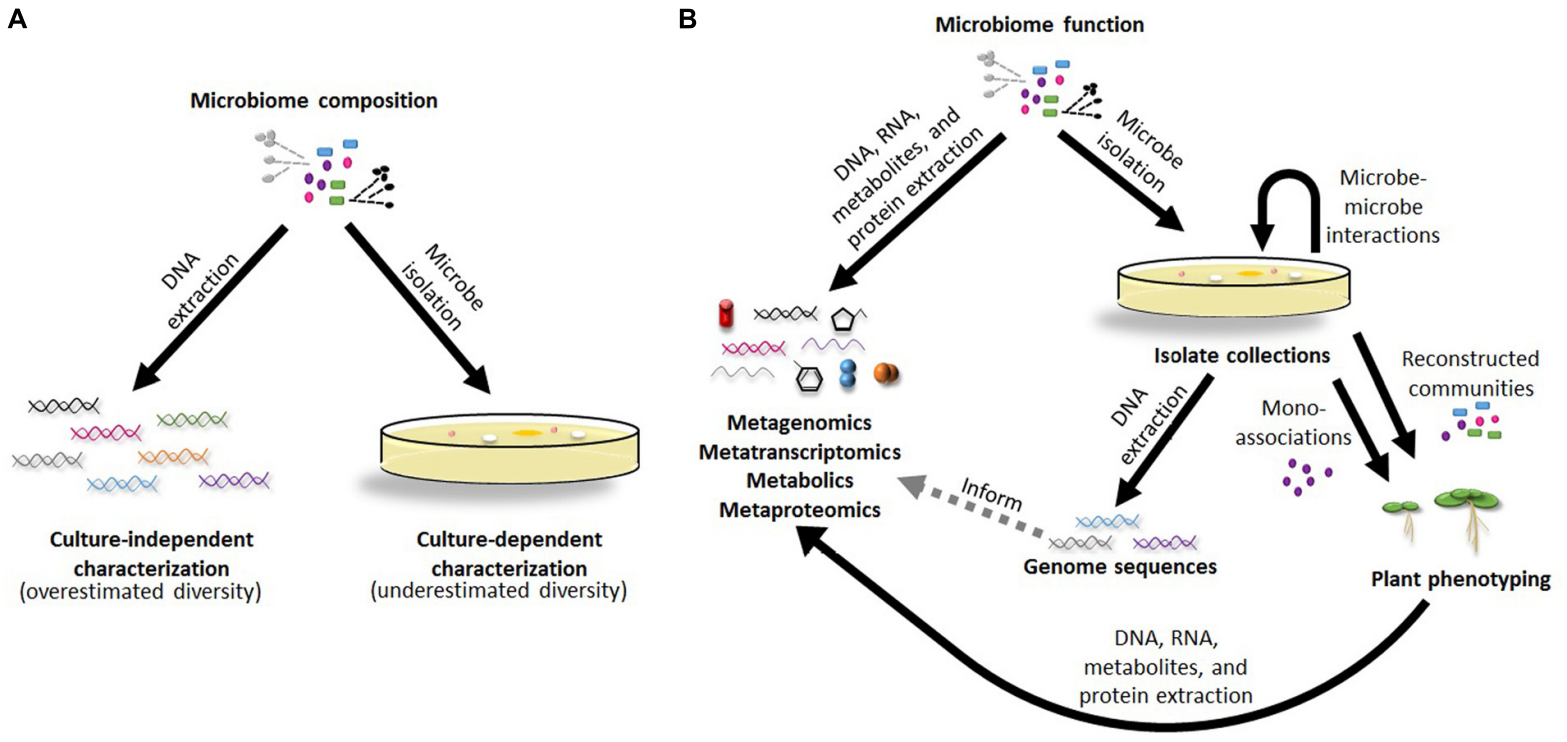
**Methods for characterization of plant microbiome composition and function. (A)** Culture-dependent and culture-independent methods are the two major approaches used to determine the microbial make-up of plant microbiomes with each having its own limitations in identifying community composition. **(B)** Proposed pipelines to integrate the data from culture-independent and culture-dependent methods together to address functions of plant microbiomes and the individual types of microbes found within them.

Initial studies to characterize plant-associated communities relied on cultivation-based methods. Although culture-dependent studies are able to make important conclusions about specific, readily isolated microbes (Bakker et al., 2013), they are biased in the taxa they identify and drastically limit community diversity estimates (**Figure 1A**; Pace, 1997). Even though these approaches give an incomplete view of microbiomes and lack the sensitivity to detect shifts in community composition, isolated microbes provide the raw material for genome sequences and determination of plant phenotypes following colonization, enabling scientists to address more complicated questions about plant microbiomes (**Figure 1B**). More recently, massive parallel sequencing has dramatically improved our ability to identify and quantify community members, even down to extremely rare taxa, unearthing insights beyond the information provided by individual microbes. These technologies use miniaturized, spatially separated clonal amplification to sequence, rather than individual Sanger sequencing reactions, with various chemistries including: pyrosequencing (e.g., 454), reverse dye terminator (e.g., Illumina), phospholinked fluorescent nucleotides (e.g., PacBio), and proton detection (e.g., Ion Torrent; Glenn, 2011). Although each platform differs in up-stream preparations and down-stream analyses, they all share the potential to detect and differentiate rare microbes in the soil, one of the most diverse environments on earth. By performing a survey of all 16S rRNA sequences present in a given community, these studies give a detailed picture in terms of diversity and community composition. Despite this, they are still only a projection of a microbial community, do not differentiate live from dead cells, and potentially contain sequencing errors that lead to misinterpretations of the data, including diversity overestimations (**Figure 1A**). While recent technical advancements use individual DNA molecule tagging in plant microbiomes to control for PCR errors and biases (Lundberg et al., 2013), these methods are not yet widely used. An ideal method to characterize plant microbiomes would provide the certainty of isolated microbes with the breadth of massive parallel sequencing.

**FIGURE 2:**
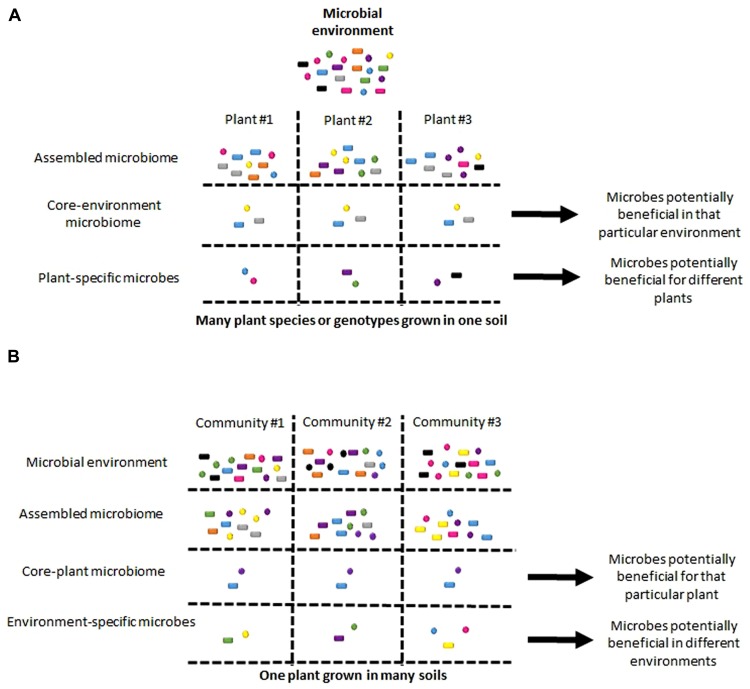
**Comparing “core” and “specific” plant microbiome members.** Potential conclusions that might be made for “core” and “specific” microbes when comparing communities from **(A)** many species/genotypes of plant grown in the same environment or **(B)** the sample plant grown in many environments. These differences in environment are not limited to the microbial inoculum, but also abiotic differences, such as nutrient levels.

Studies using massive parallel sequencing have defined plant-associated microbial communities for a wide variety of plant species from as small as the model species *Arabidopsis thaliana* (Bulgarelli et al., 2012; Lundberg et al., 2012) to as large as trees (Redford and Fierer, 2009; Redford et al., 2010; Gottel et al., 2011), even for a number of crops including: corn (Peiffer et al., 2013), lettuce (Schreiter et al., 2014), potato (Inceoglu et al., 2011), and rice (Sessitsch et al., 2012). Although it is difficult to make direct comparison of all of these results due to the various sequencing protocols used, in *A. thaliana* studies differentiating between the external rhizosphere and internal root bacterial communities, it is clear that soil, rhizosphere, and root form three distinct communities (Lundberg et al., 2012), corresponding to their different microenvironments (Bakker et al., 2013). Notably, rhizosphere communities are more similar to their soil inoculum than to internal root communities in terms of diversity and composition, with both bacterial load and diversity being lower in root communities (Bulgarelli et al., 2012; Lundberg et al., 2012). When root microbiome composition was determined for four different species within a plant family (*A. thaliana*, *A. lyrata*, *A. halleri*, and *Cardamine hirsuta*) grown in the same soil, the community differences could not be fully explained by plant phylogeny (Schlaeppi et al., 2013), leaving room for the other influencing factors such as microbe–microbe interactions. Further, the composition of these root microbiomes also share a set of “core” members within the phyla of Actinobacteria, Bacteroidetes, and Proteobacteria, implying there are conserved mechanisms of colonization across ~25–30 million years of separation between plant species (**Figure 2A**; Schlaeppi et al., 2013). In contrast, no “core” community was observed in the phyllosphere samples of 56 diverse trees grown in the same location (Redford et al., 2010). This difference may be due to the number of plant species examined, the genetic diversity among the trees observed, or the inherent differences between colonization of roots by soil microbes and leaves by air-borne microbes (Maignien et al., 2014). When the same plant is grown in different soils, there is remarkable similarity between microbial communities. For *A. thaliana* studies, the microbes harbored in internal root communities grown in four different soils from two different continents had phyla distributions with universal increases in certain microbes, such as Actinobacteria, and decreases in other microbes, such as Acidobacteria compared to the rhizospheres and soils (Bulgarelli et al., 2012; Lundberg et al., 2012). For maize rhizosphere communities grown in five diverse fields, although communities can be differentiated, the actual rhizosphere microbiome compositions look remarkably alike (Peiffer et al., 2013). In phyllosphere communities, which are excellent to studies succession dynamics (Redford and Fierer, 2009), there is minimal impact of geographic distribution up to thousands of kilometers in *Pinus ponderosa* needle microbiomes (Redford et al., 2010) and between growing seasons of deciduous leaf microbiomes (Redford and Fierer, 2009). Together, these results suggest that a core microbiota can be recruited from very diverse microbial surroundings, narrowing down both the most relevant community members and pointing to the host detriments controlling the mechanisms of assembly (**Figure 2B**). Interestingly, early colonization events set up alternative community composition outcomes in phyllosphere communities (Maignien et al., 2014), suggesting that beyond selective pressures imposed by the host, microbe–microbe interactions and/or random environmental effects may contribute to plant microbiome composition. These studies provide a more detailed picture of plant microbiome compositions, which gives the opportunity to determine the underlying pathways of community assembly.

## POTENTIAL MODULATION OF PLANT MICROBIOME COMPOSITION BY THE PLANT IMMUNE SYSTEM

The consistency seen between different plant microbiomes generated from multiple microbial environments strongly implies that host determinants provide selective pressure during community assembly. The focus here is on how the plant immune might function to whittle down microbiome candidates, helping to create a robust subset of microbes present in plant-associated microbial communities. Immune systems of all organisms function to recognize “self” from “non-self” and normally maintain self-homeostasis by destroying “non-self.” While the mechanisms by which pathogens and beneficial microbes trigger and evade the plant immune system are well-defined (Zamioudis and Pieterse, 2012; Dangl et al., 2013), how the immune response promotes health and homeostasis through assembling a service-providing, “non-self” microbiome is still being determined. A variety of microbes can activate the plant immune system at the level of both extra- and intracellular receptors, initiating subsequent signal transduction (Torres and Dangl, 2005; Fujita et al., 2006; Cramer et al., 2011). These responses have been characterized mostly in leaf tissue, but also occur in the roots (Millet et al., 2010), and include: programmed cell death, cell wall thickening, antimicrobial compounds expression, reactive oxygen species generation, and defense phytohormones production (Baron and Zambryski, 1995; Torres and Dangl, 2005). These small molecule hormones, particularly salicylic acid (SA), jasmonic acid (JA), and ethylene, are a class of plant secondary metabolites that coordinate cellular responses to biotic and abiotic stresses and integrate induction of immune system output responses (Pieterse et al., 2012). Thus, it is understandable that suppression of the host immune system is a common target to facilitate colonization, not only for pathogens, but also for beneficial microbes.

Because the plant defense hormones are central integrators, their biosynthesis or perception are logical places to begin analysis of how the plant immune system effects microbial colonization at the community-level. At the isolate-level, SA is locally suppressed to facilitate colonization by two classical examples of mutualistic microbes, arbuscular mycorrhizal (AM) and species of *Rhizobia* (Garcia-Garrido and Ocampo, 2002; Stacey et al., 2006). These results suggest SA signaling provides sufficient selective pressure to warrant microbial avoidance/interference strategies. To date, plant microbiome studies on *A. thaliana* with SA mutants or treatments have relied on culture-dependent methods of measuring microbial species richness, diversity, and load (Kniskern et al., 2007; Doornbos et al., 2011), making it difficult to observe the subtle community differences only seen in the culture-independent studies. Further, although culture-dependent and -independent studies do not show a consistent impact of JA signaling on phyllosphere or root microbiome composition (Kniskern et al., 2007; Doornbos et al., 2011; Santhanam et al., 2014), recent massive parallel sequencing studies identified ethylene signaling contributions to *A. thaliana* phyllosphere microbiome composition (Bodenhausen et al., 2014). Together, these results represent the start of controlled experiments taking advantage of isogenic mutants in *A. thaliana* to understand how phytohormones might impact the composition of plant-associated microbial communities.

Beyond these community survey approaches, metatranscriptomic and metagenomic studies have also begun to reveal possible involvement of the immune system in mechanisms of microbiome assembly. Recently, time course studies connect changes in *A. thaliana* rhizosphere communities at four developmental stages with differences in the composition of root exudates at those times (Chaparro et al., 2013, 2014). While a number of different compounds are present in root exudates, this correlation focuses on the levels of sugars and phenolic compounds, such as SA, which aid in immunity against pathogens (Clay et al., 2009; Millet et al., 2010). Hence, seedlings produce more sugars and less phenolics, while plants that are bolting or flowering secrete more phenolics and less sugars in their exudates (Chaparro et al., 2014). These results suggest at least two stages to rhizosphere assembly: early non-specific recruitment and later community selection. Further, metagenomic studies in rice found endophytic root bacteria contain several groups of genes involved in: motility, plant polymer degradation, iron acquisition (e.g., siderophores), quorum-sensing, and detoxification of reactive oxygen species (Sessitsch et al., 2012), indicating control over those pathways is important for colonization by root microbiome members. These studies represent how new sequencing technologies reveal insights into the involvement of the plant immune system during microbial colonization of the rhizosphere and root tissue.

## SERVICES PROVIDED TO THE PLANT BY ITS MICROBIOME

Several examples of soil borne beneficial microbes have been well characterized as plant growth promoting rhizobacteria (PGPR) and Fungi PGPF to produce plant growth promoting hormones (e.g., auxin), improve host nutrition, and protect plants from both abiotic and biotic stresses (Pozo and Azcon-Aguilar, 2007; Berendsen et al., 2012). Classic examples for mutualistic microbes include *Rhizobia* spp. and AM that exchange plant carbohydrates for nitrogen fixation and phosphate mobilization services for the plant (Spaink, 2000, Harrison, 2005). Another interesting example of nutrient-related beneficial microbes are *Sphingomonas* strains that can out-compete the pathogen *Pseudomonas syringae* for the same nutrients on *A. thaliana* leaves (Innerebner et al., 2011); thus, providing the plant protection. Beyond nutrient-based services, PGPR and PGPF can also induce immune “priming,” which does not refer to direct immune activation, but rather an acceleration of subsequent defense responses to pathogens (Conrath, 2006), even in distal tissues. Thus, protective rhizobacteria trigger induced systemic resistance (ISR) and AM can produce mycorrhizal induced resistance (MIR; Pozo and Azcon-Aguilar, 2007; Zamioudis and Pieterse, 2012) each in a JA-dependent manner (Pieterse et al., 1998; Pozo and Azcon-Aguilar, 2007; Pozo et al., 2008; Van der Ent et al., 2009), suggesting that microbial exploitation of this phytohormone pathway is common. Recently, the ability of *P. fluorescens* WCS417 to promote increased leaf and root biomass was separated from its ISR triggering in *A. thaliana* (Zamioudis et al., 2013). Thus, it was demonstrated that stimulation of lateral root and root hair development occurs via an auxin-dependent and JA-independent mechanism (Zamioudis et al., 2013). The interactions between PGPR/PGPF and their plant host illustrate the power to unravel mechanisms with isolated microbes, and indicate involvement of the immune system in both assembly and function of root-associated microbes.

Detailed mechanistic pathways from PGPR and PGPF provide expected results for metatranscriptomic and metagenomic studies of plant microbiomes. Thus, in a study on rice roots combining metagenome and 16S rRNA sequence analysis with targeted qPCR of bacterial RNA, several transcripts related to nitrogen cycling that had previously been characterized in *Rhizobia* spp. studies (Spaink, 2000) were correlated with predicted *Rhizobia* spp. in these communities, suggesting nitrogen fixation is a service is provided in a community context, not just Rhizobia spp. in mono-association with plants (Sessitsch et al., 2012). Further, it is possible that comparison between these types of studies with 16S ribotyping data will demonstrate that taxonomically diverse microbes may be functionally redundant (Burke et al., 2011; Lozupone et al., 2012), highlighting the importance of using multiple approaches on the same samples (**Figure 1B**).

## PLATFORMS FOR FUTURE PLANT MICROBIOMES STUDIES

Currently, many plant microbiome studies exist in mostly disjointed pieces. In essence, we have isolate collections with known functional mechanisms outside of their whole community context in one hand and projected communities with unclear functions in the other. Despite advances in high-throughput sequencing technologies, it is still challenging to answer questions regarding microbiome function due to lack of experimental microbiome genetic/functional characterization. Thus, although sequencing costs continue to decrease making metagenomic, metatranscriptomic, metabolic, and metaproteomic approaches increasingly available, the good quality reference databases they rely on need to likewise improve (Ye et al., 2013). Ultimately, I believe one solution for this problem could be in partnering the culture-independent studies with the culture-dependent ones (**Figure 1B**). For this to happen, we need to build matching isolate collections from communities with defined 16S rRNA plant microbiome composition, allowing the further dissection of the nature of interactions between plants and their symbiotic communities, and the interactions of these microbes with each other. These collections also have the potential to be developed as cocktails of microbial probiotics for plant health in agronomic uses.

The need for plant microbiome isolate collections also extends to the determination of community composition for previously uncharacterized types of microbes. Even with the well-characterized bacterial ribosomal database, it is clear the 16S rRNA gene is insufficient to decipher between closely related strains that might have very different phenotypic output, potentially leading to very misleading conclusions about the community. For example, the genus *Pseudomonas* contains examples of strains ranging from PGPR to pathogens, which depending on the length and region of 16S rRNA gene sequenced may appear the same (Blakney and Patten, 2011). Although approaches using conserved genes other than 16S rRNA gene are encouraging (Sunagawa et al., 2013), examining multiple loci simultaneously is needed to overcome the fundamental limitations of single marker profiling.

In order to create useful databases for both metagenomic and multiple marker profiling studies, significant effort must be given to the isolation as many root- or leaf-enriched microbiota defined by ribotyping censuses as possible. The projected phyla distributions in these communities (Bulgarelli et al., 2012; Lundberg et al., 2012; Maignien et al., 2014) contain a higher percent of cultivable bacteria than soil or rhizosphere communities (Reinhold et al., 1986; Barraquio et al., 1997; Tringe et al., 2005), such as Actinobacteria, Bacteroidetes, Firmicutes, and Proteobacteria. Possessing comprehensive and cataloged collections of cultured plant-associated microbiota offers the opportunity for plant phenotyping and genome sequencing to further address underlying microbiome functions. This has already been accomplished in many plant species with notable examples in *Populus deltoidetes* (Brown et al., 2012). Further, efforts to create isolate collections and perform preliminary phenotyping provide the plant microbiome field the opportunity for collaboration between research and undergraduate education, allowing the next generation of scientists to contribute to our scientific community in exchange for engaging, interdisciplinary training (Bascom-Slack et al., 2012). Such collections could ultimately lead to construction of synthetic communities of individually traceable, known microbial inputs, decreasing the noise inherent to complex communities while allowing the testing of well-studied principles of plant–microbe mono-associations within a community context. From such experiments, quantitative information provides high confidence information of the exact microbiome membership for each plant, and if the genomes of the community members are known, the full metagenome can also be inferred from the set of genes detected (Goodman et al., 2009; Faith et al., 2011, 2013, 2014; McNulty et al., 2013; Rey et al., 2013). Collections of plant microbiome isolates also facilitate unraveling the impact that microbes have on each other’s growth within these communities. For example, this role is potentially important for internal *A. thaliana* root communities, which harbor a large proportion of Actinobacteria (Bulgarelli et al., 2012; Lundberg et al., 2012), a phyla with diverse genera and ability to produce an array of secondary metabolites, such as antibiotics (Raaijmakers and Mazzola, 2012). These developments, and their use in controlled experimental conditions, are imperative to allow the eventual development of robust and resilient microbial cocktails that can perform useful ecosystem services for plants in agronomic settings. Together, the recent developments in this field provide the opportunity to understand how the immune system plays in mediates the relationships between the plant microbiome and host health.

## Conflict of Interest Statement

The author declares that the research was conducted in the absence of any commercial or financial relationships that could be construed as a potential conflict of interest.
